# Analysis of differentially expressed *Sclerotinia sclerotiorum* genes during the interaction with moderately resistant and highly susceptible chickpea lines

**DOI:** 10.1186/s12864-021-07655-6

**Published:** 2021-05-08

**Authors:** Virginia W. Mwape, Fredrick M. Mobegi, Roshan Regmi, Toby E. Newman, Lars G. Kamphuis, Mark C. Derbyshire

**Affiliations:** 1 Centre for Crop and Disease Management, Curtin University, Bentley, WA 6102 Australia; 2 Commonwealth Scientific and Industrial Research Organization, Agriculture and Food, Floreat, WA Australia

**Keywords:** *Sclerotinia sclerotiorum*, *Cicer arietinum*, CAZymes, Secondary metabolites, Secreted effectors, Transcription factors, Infection

## Abstract

**Background:**

*Sclerotinia sclerotiorum*, the cause of Sclerotinia stem rot (SSR), is a host generalist necrotrophic fungus that can cause major yield losses in chickpea (*Cicer arietinum*) production. This study used RNA sequencing to conduct a time course transcriptional analysis of *S. sclerotiorum* gene expression during chickpea infection. It explores pathogenicity and developmental factors employed by *S. sclerotiorum* during interaction with chickpea.

**Results:**

During infection of moderately resistant (PBA HatTrick) and highly susceptible chickpea (Kyabra) lines, 9491 and 10,487 *S. sclerotiorum* genes, respectively, were significantly differentially expressed relative to in vitro. Analysis of the upregulated genes revealed enrichment of Gene Ontology biological processes, such as oxidation-reduction process, metabolic process, carbohydrate metabolic process, response to stimulus, and signal transduction. Several gene functional categories were upregulated *in planta*, including carbohydrate-active enzymes, secondary metabolite biosynthesis clusters, transcription factors and candidate secreted effectors. Differences in expression of four *S. sclerotiorum* genes on varieties with different levels of susceptibility were also observed.

**Conclusion:**

These findings provide a framework for a better understanding of *S. sclerotiorum* interactions with hosts of varying susceptibility levels. Here, we report for the first time on the *S. sclerotiorum* transcriptome during chickpea infection, which could be important for further studies on this pathogen’s molecular biology.

**Supplementary Information:**

The online version contains supplementary material available at 10.1186/s12864-021-07655-6.

## Background

*Sclerotinia sclerotiorum* is a necrotrophic fungal pathogen with a remarkably broad host range of over 600 plant species [1, 2]. The hosts of *S. sclerotiorum* include economically important crops such as *Brassica napus* (canola), *Glycine max* (soybean), *Phaseolus vulgaris* (common beans), *Pisum sativum* (field pea), *Helianthus annuus* (sunflower) and *Cicer arietinum* (chickpea) [1]. Research on genetic and molecular management of various fungal pathogens in chickpeas, such as *Aschochyta rabiei* and *Fusarium oxysporum* f. sp. *ciceris*, has led to the identification of genetic and pathological variabilities leading to shifting from cultural practices to the development of new genetic and molecular management approaches [3]. However, limited information is available on the molecular biology of *S. sclerotiorum* during chickpea infection, despite the fact that, in a conducive environment, disease caused by *Sclerotinia* species can cause up to 100% chickpea yield loss [4, 5].

*S. sclerotiorum* is generally described as a necrotroph. As such, it derives its energy from dead plants to complete its lifecycle; this contrasts with biotrophs, which feed on living plant cells. However, recent studies indicate that *S. sclerotiorum* undergoes a brief biotrophic phase soon after penetration [6]. Expression of biotrophy-related genes, including those with Lysin Motif (LysM) domains, within the first 24 h post-inoculation (hpi) during the *S. sclerotiorum - B. napus* interaction has been reported [7]. Furthermore, previous studies have shown that *S. sclerotiorum* integrin-like protein (*SSITL*) and chorismate mutase (*SsCm1*) may suppress host defence signalling during the biotrophic phase [6–8]. The pathogenesis journey through the two phases requires regulation of metabolic, virulence and defence enzymes in response to challenges associated with the type of host tissue, nature of energy source, acidity, and oxidative stress [9, 10].

The *S. sclerotiorum* reference genome has revealed several potential pathogenicity and virulence factors, including cell wall degrading enzymes (CWDES), metabolites, detoxification enzymes and candidate secreted effectors [11–13]. We refer to pathogenicity factors as genes that are essential for causing disease and virulence factors as genes that contribute in a quantitative manner to pathogen aggressiveness; any genes that have an impact on growth away from the plant host are referred to in this article as ‘developmental factors’, and these may also be pathogenicity or virulence factors at the same time [14–16]. Amselem et al. [13] compared the genomes of *S. sclerotiorum* and its relative *B. cinerea* and found a variety of putative secreted enzymes, including carbohydrate-active enzymes (CAZymes) such as xylanases, pectinases, polygalacturonases (PGs), hemicellulases, and cellulases. CAZymes play a crucial role in host cell wall degradation to simpler monomers that serve as a carbon source [17]. Disruption of the *S. sclerotiorum* CAZymes arabinofuranosidase/β-xylosidase and an endo-β-1, 4-xylanase showed reduced or lost virulence [18], an indication of their importance in the growth and virulence of the pathogen.

Secreted effector candidates have also been found in *S. sclerotiorum*. These are proteins that manipulate host cell functions and suppress plant defence to promote infection [13]. Some of these candidates have been functionally characterised. For example, secreted protein *SsSSVP1* manipulates plant energy metabolism for full virulence [19]. Disruption of *SsSSVP1* in *S. sclerotiorum* significantly reduces virulence in *B. napus* and *Arabidopsis thaliana*, compared to the wild type [19]. *S. sclerotiorum* strains lacking *SSITL* cause rapid induction of plant defence genes associated with the salicylic acid and jasmonic acid/ethylene signalling pathway, suggesting *SSITL* as a possible effector that plays a key role in suppressing host immunity at an early stage of infection [6, 20].

Transcription factors (TFs) act as pivotal regulators of gene expression by binding to gene promoters to activate or repress expression [15]. Several *S. sclerotiorum* transcription factors have been characterised. For example, in response to reduced acidity, the *S. sclerotiorum* gene encoding a zinc finger transcription factor (*Pac1*) triggers oxalic acid (OA) biosynthesis, causing an increase in expression of exo-polygalacturonase (*Sspg1*), which is involved in pectin degradation, a significant constituent of the plant cell wall [21]. Although not directly involved in pathogenicity, *Pac1* plays a role in OA and *Sspg1* accumulation.

Recent studies of *S. sclerotiorum* gene expression on different hosts found that a gene encoding oxaloacetate acetylhydrolase (*Ssoah1*), known to be vital for OA production, was expressed in a similar pattern during infection of *B. napus* [5, 17] and *P. vulgaris* [22]. However, *Ssoah1* expression was not observed during *G. max* infection [23]. Intrinsic host immunity may also affect the pattern of *S. sclerotiorum* gene expression as demonstrated in *B. napus,* where a gene encoding a polygalacturonase, *Sspg1*, was upregulated in a resistant variety, with no upregulation in a susceptible variety relative to in vitro [24]. These discrepancies indicate that *S. sclerotiorum* gene expression may depend on the host species and intraspecific differences in levels of resistance.

Our study aimed to (1) understand further how the *S. sclerotiorum* transcriptome is deployed *in planta* relative to in vitro conditions; (2) catalogue upregulated and downregulated genes in the *S. sclerotiorum* - chickpea pathosystem; and (3) evaluate the differences in gene regulation during *S. sclerotiorum* infection of a moderately resistant and a susceptible chickpea line. The current study hypothesised that (i) *S. sclerotiorum* would deploy an array of factors to facilitate chickpea infection and (ii) *S. sclerotiorum* will express genes that are specific to moderately resistant and susceptible varieties. This study reveals the activation of primary *S. sclerotiorum* pathogenesis factors, including CAZymes and affiliated proteins, putative secreted effector proteins, secondary metabolites and genes involved in regulating production of and tolerance to reactive oxygen species (ROS) such as catalases and peroxidases.

## Results and discussion

### Processing and filtering of transcriptome data

RNA-seq was used to compare *S. sclerotiorum* gene expression between samples taken during infection of two *C. arietinum* lines and during growth in vitro. Between 1.8 to 61.8% of sequence reads derived from the infected moderately resistant (MR) line samples, which were collected between 6 and 72 hpi, mapped to the reference genome of *S. sclerotiorum*. On the other hand, between 0.7 to 68.1% of sequence reads derived from infected susceptible line samples collected between 6- 72hpi mapped back to the *S. sclerotiorum* genome (Table 1). At 72 hpi, the average percentage of reads mapping to the fungal genome in the S line was higher (68.1%) than in the MR line (61.8%), suggesting that the S line tissues may be more heavily colonised than those of the MR line (Table 1). The larger lesions found on the S line at the later stage of infection during the current study (results not shown) and greater abundance of fungal RNA in the S line samples together suggest that it exhibited greater levels of fungal colonisation than the MR line. Such differences have been reported in previous *S. sclerotiorum* transcriptome studies [5, 18, 19, 23].

**Table 1:** Summary of the Illumina sequence reads generated by RNA – seq obtained from inoculation of a moderately resistant (MR) chickpea line PBA HatTrick and a susceptible (S) chickpea line Kyabra. The values for each time point are the averages of the three biological replicates

Host	Hours post inoculation (hpi)	Total raw read pairs	Trimmomatic reads retention (%)	BBSplit reads separation	
				***S. sclerotiorum***	***C. arietinum***
MR	6	67,354,385	98.7	1,207,201 (1.8%)	66,147,184 (98.2%)
	12	68,680,857	98.76	5,929,006 (8.6%)	62,751,851 (91.4%
	24	61,114,985	98.91	28,078,637 (45.9%)	33,036,348 (54.1%)
	48	56,616,306	98.85	40,566,767 (71.7%)	16,049,538 (28.3%)
	72	63,109,260	98.92	39,012,318 (61.8%)	24,096,941 (38.2%)
S	6	58,025,893	98.4	414,371 (0.7%)	57,611,521 (99.2%)
	12	72,896,961	98.4	1,851,043 (2.5%)	71,045,918 (97.5)
	24	54,049,381	98.4	18,414,027 (31%)	35,635,354 (65.9%)
	48	60,727,165	98.5	36,714,863 (60.4%)	24,012,301 (39.5%)
	72	57,636,636	98.5	39,273,084 (68.1%)	18,363,551 (31.9%)
				**20, 961,027	**40,875,050
In vitro	0	56,566, 082	96.8	53,907,476 (95.3%)	NA

**averages number of reads

**Fig. 1:**
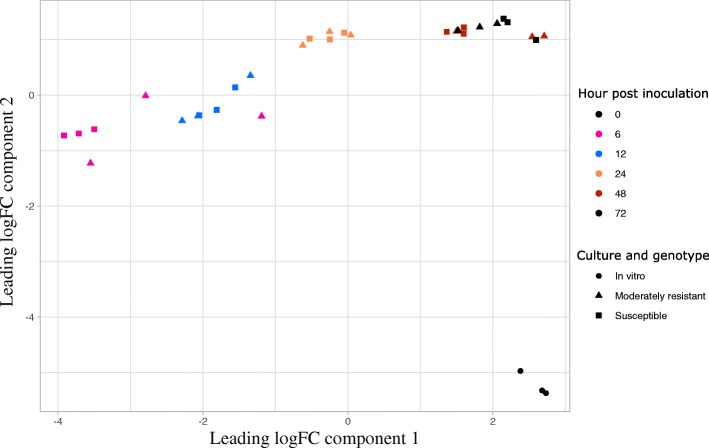
A multidimensional scaling (MDS) plot showing the relatedness of *Sclerotinia sclerotiorum* samples used for RNA-Seq analysis. Samples were collected from moderately resistant (MR) and susceptible (S) chickpea lines at 6, 12, 24, 48 and 72 h post inoculation (hpi), as well as samples from an in vitro culture. The symbol ▲represent the MR, ■ the S and ● the in vitro samples. The x and y-axis represent Euclidean dimensions, distinct colours represent each treatment, and individual dots represent each sample

The similarity of the three biological replicates and the accuracy of the RNA-seq analysis was demonstrated using classic multidimensional scaling (MDS), which shows the MDS plot of distances between gene expression profiles (Fig. 1). The MDS showed a distinct grouping of samples grown in vitro and *in planta* at the early (6–12 hpi), the mid (24 hpi) and late (48–72 hpi) stage of infection (Fig. 1). There was a clear distinction between the *S. sclerotiorum* transcriptomes at 24 and 48–72 hpi, an indication of the significant differences in the types of genes expressed at these time points.

### Validation of RNA-seq data using reverse transcription-quantitative PCR

**Fig. 2:**
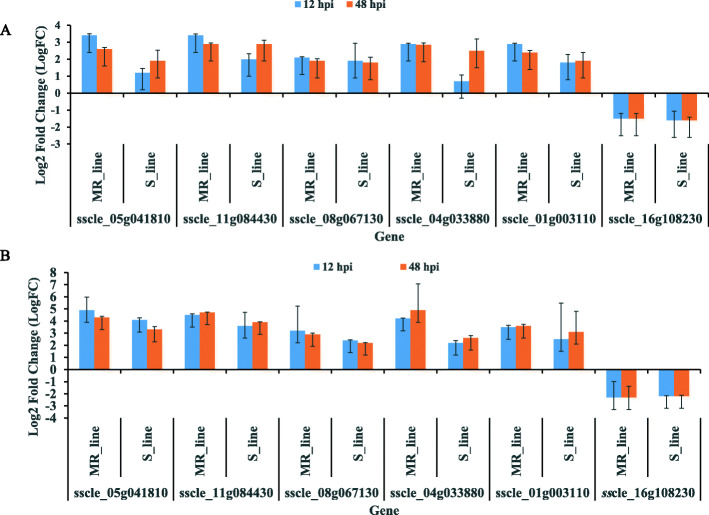
Reverse transcription**-**quantitative PCR (RT-qPCR) validation of RNA sequencing (RNA-Seq) data in the moderately resistant (MR) and susceptible (S) chickpea lines following infection with *Sclerotinia sclerotiorum*. Log_2_(fold change) (LogFC) values were generated for qPCR samples by comparing the expression of genes at each time point of infection vs the in vitro control sample using the 2 ^-∆∆Ct^ method (**a**). LogFC values were generated for RNA-Seq samples by comparing the average raw read counts at each time point of infection vs in vitro/vegetative growth culture (**b**). Pairwise contrasts were performed using quasi-likelihood F tests. The data are presented as means ± standard error (SE) from three biological replicates for 12 hpi (early stage of infection) and 48 hpi (late stage of infection)

To validate the accuracy of the RNA-seq data, five upregulated genes and one downregulated gene in both chickpea lines at 12 hpi (early infection stage) and 48 hpi (late infection stage) were quantified using reverse transcription quantitative polymerase chain reaction (RT-qPCR) (Fig. 2). Six genes of which, according to RNA-seq analysis, five were significantly upregulated (sscle_05g041810, sscle_11g084430, sscle_08g067130, sscle_04g033880 and sscle_01g003110) and one was significantly downregulated (sscle_16g108230) were randomly selected for validation. These genes, their putative functions and the primer sequences are listed in Table S1. The expression patterns for each gene in our qPCR assay (Fig. 2a) were similar to the expression observed in the RNA-seq data (Fig. 2b). These results thus show a correlation between our qPCR and RNA-seq data.

### Genotype-specific and genotype non-specific differential gene expression during *Sclerotinia sclerotiorum* infection of chickpea

**Fig. 3:**
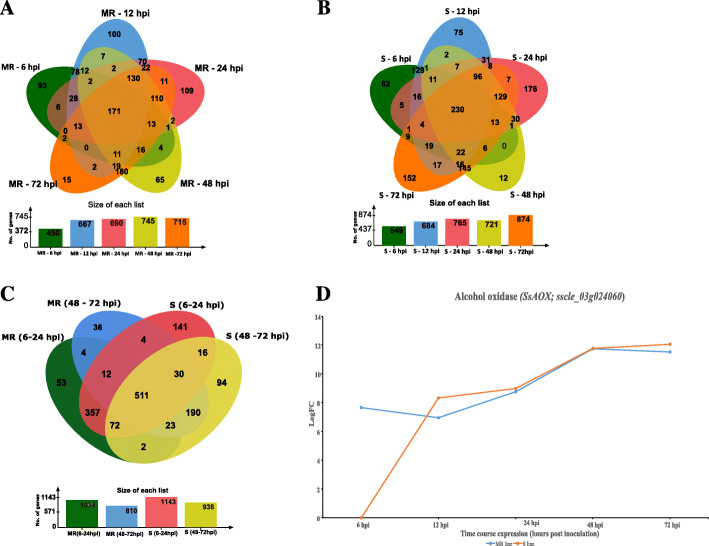
Venn diagram and graph showing upregulated *Sclerotinia sclerotiorum* genes during interaction with chickpea. Venn diagram shows the number of common and unique genes at time points 6, 12, 24, 48, and 72 hpi in (**a**) moderately resistant (MR), and (**b**) susceptible (S) lines (**c**) Comparison of MR and S genes (**d**). A graph showing expression pattern during the time course of infection of the most highly expressed common gene between MR and S line

Based on the distinct differences between *the in planta* and in vitro samples demonstrated in the MDS plot (Fig. 1), we expected that many *S. sclerotiorum* genes would be differentially expressed *in planta* relative to in vitro, irrespective of the susceptibility level of the host line. Therefore, we first assessed whether there were significant differences in read counts for each of the infection time points for each host relative to in vitro. We identified upregulation of 2150 and 3593 and downregulation of 7341 and 6894 *S. sclerotiorum* genes during MR and S line infection, respectively (Fig. 3a and b, Table S2, Figure S1). There were 171 common genes upregulated in MR line (Fig. 3a) and 230 common genes upregulated in S line (Fig. 3b). A comparative analysis of the upregulated genes between the MR and S genotypes during the early stage (6–12 hpi) and late stage (48–72 hpi) of infection revealed that 511 genes were differentially expressed relative to in vitro at the same time points on both the MR and S lines (Fig. 3c). A gene encoding an alcohol oxidase (*SsAOX*; sscle_03g024060) was the most upregulated gene common to the two chickpea genotypes (Fig. 3d). An alcohol oxidase in *Cladosporium fulvum* has been suggested to be a key component in the detoxification of antifungal compounds released from the plant cell wall during infection [25]. Similarly, two putative hydrophobic cell surface proteins (sscle_12g091650 (logFC = 9.6–12.5) and sscle_09g070510 (LogFC = 7.3–8.6) were the most highly upregulated at an early stage of infection relative to in vitro across both varieties. The gene sscle_12g091650 contains a hydrophobic surface binding protein A (HsbA) domain (PF12296) which was originally identified in *Aspergillus oryzae* as a surface protein that plays a key role in both the adhesion to and degradation of hydrophobic surfaces [26]. Similarly, sscle_09g070510 contains a repeated fasciclin domain (PF02469) which has been reported in *Magnaporthe oryzae* to be important in adhesion and binding to hydrophobic surfaces [27]. Our findings suggest these two genes might have a role during the *S. sclerotiorum* biotrophic phase during chickpea infection.

The current study describes genes upregulated in both the MR and S lines when compared to in vitro (Table S3). Comparing the transcription changes in the MR and S lines showed that there were also differences between lines in expression of some *S. sclerotiorum* genes relative to in vitro, with 82 and 251 genes upregulated exclusively in the MR or S line, respectively (Figure S2, Table S4). There were 42 genes with functional domains expressed either in the MR or S line only and these are involved in cell wall degradation, secondary metabolite biosynthesis, transport, detoxification, and signalling (Figure S2).

The common genes and these exclusively upregulated genes are discussed in various sections below. To note are two genes upregulated in the MR only which are involved in sugar glucose and carboxylate catabolism, metabolism and anabolism (sscle_01g005580 and sscle_05g040510) (Figure S2), indicating the importance of hydrolytic activities during infection of chickpea. Previous research has found pentose phosphate is critical in fungal pathogens for supplying cells with NADPH for detoxification of ROS and virulence [27, 28]. A gene involved in the pentose-phosphate pathway (sscle_01g005580) was upregulated in the MR line only. The full virulence of *S. sclerotiorum* requires detoxification of ROS, an important component of the host defence response [29], suggesting that *S. sclerotiorum* upregulation of sscle_01g005580 may be a managing strategy of host resistance responses.

Expression analysis of the MR versus S line at each time point showed that genes with different expression relative to in vitro in the two lines (, there were only four genes that were differentially expressed between genotypes at any given time point (Table S2). This included two genes downregulated in the MR relative to the S line (upregulated in the S line) at 6 hpi and the other two upregulated in the MR relative to the S line (downregulated in S line) at 48 hpi. The genes sscle_09g073140 (logFC = 5.1, p_adj_ = 0.02) and sscle_04g033530 (log FC = 4.2, p_adj_ = 0.04) were differentially expressed at 6 hpi and sscle_16g111070 (logFC = 5.3, p_adj_ = 0.004) and sscle_05g047520 (logFC = 5.3) were differentially expressed at 48 hpi. These four genes are predicted in the *S. sclerotiorum* genome, but they have no known functional domains. Therefore, it is not possible to speculate much on their role during specific interactions between MR and S chickpea genotypes.

We also performed an analysis where we included the genotype x timepoint interaction. The final design as a factor and found that this interaction was not significant for any genes (P_adj_ = 0.05), suggesting that all genes had temporally similar expression patterns between the two lines. We did not include hosts (*C. arietinum*) differentially expressed genes in the current manuscript, as this will form a discrete study along with other data in future. However, the limited differences in expression of *S. sclerotiorum* genes between the two hosts would suggest that they present a qualitatively similar environment to the pathogen despite one of them, the MR line, reducing the extent of pathogen growth.

### Gene ontology term enrichment analysis of upregulated genes identifies multiple biological and molecular functions associated with infection

Gene Ontology (GO) enrichment analysis is a powerful technique for analysing differential gene expression data to gain insight into the broader biological processes (BP), molecular functions (MF) and cellular components (CC) of genes. The upregulated genes were significantly enriched with wide range of GO categories (Table S5, Figure S3). The significant categories included those involved in oxidation-reduction process (GO:0055114), proteolysis (GO:0006508), organic substance metabolic process (GO:0071704), and metabolic process (GO:0008152). GO enrichment analysis also showed significant enrichment of downregulated genes with wide range of GO categories including those involved in transmembrane transport (GO:0055085), oxidoreductase activity (GO:0016491), drug metabolic process (GO:0017144), and N-acyltransferase activity (GO:0016410) (Table S6, Figure S4).

The BPs highly enriched in the significantly upregulated set of genes, during the early stage of infection, included oxidation-reduction process (GO:0055114), protein metabolic process (GO:0019538), proteolysis (GO:0006508), cellular response to stimulus (GO:0051716) signal transduction (GO:0007165), carbohydrate metabolic process (GO:0005975) and metabolic processes (GO:0008152) (Table S5). Early defence of *Aschochyta rabiei* in chickpea has been associated with a strong accumulation of reactive oxygen species (ROS) in resistant chickpea cultivars compared to susceptible chickpea cultivars [30]. Similarly, previous research found *A. thaliana* enhanced host ROS increased resistance to *S. sclerotiorum*, and co-ordinately *S. sclerotiorum* genes involve in response to oxidative stress were overexpressed [31]. The BP category oxidation-reduction process (GO:0055114) was highly enriched exclusively in genes upregulated in the MR line at 6 hpi and 48 hpi, suggesting that *S. sclerotiorum* may focus on regulating the environment redox status during MR line infection to counter host resistance responses.

GO term enrichment analysis also provided an insight into the temporal aspects of the *S. sclerotiorum*-chickpea interaction. Genes involved in cellular communication (GO:0007154), signalling (GO;0023052), response to stimulus (GO:0050896), and signal transduction (GO:0007165) (Table S5, Figure S3) were enriched in genes upregulated in both lines at the early stage of infection (6–24 hpi; Fig. 3c), indicating the importance of rapid adaptation to *in planta* growth. Among genes upregulated in both lines at the late stage of infection (48–72 hpi; Fig. 3c), the enriched GO categories included carbohydrate metabolic process (GO:0005975), and metabolic process (GO:0008152) (Table S5, Figure S3) among others, an indication of the importance of utilisation of energy sources during the necrotic phase of *S. sclerotiorum* infection. The most significantly enriched GO categories in the current study grouped into carbohydrate-active enZYmes (CAZymes), proteases, transporters, transcription factors and other secondary metabolites. Genes were categorised based on their functions and predicted roles to simplify the study, as discussed below.

### Genes involved in the degradation of the host cuticle

The plant cuticle is the first physical barrier to pathogen invasion and is composed of lipid-derived polyester and cuticular waxes [32]. In the current study, *S. sclerotiorum* genes encoding cutinases and lipases were upregulated throughout infection. Interestingly, four *S. sclerotiorum* genes encoding lysophospholipase (sscle_02g020060), carboxylesterase (sscle_03g027590), GDSL-lipase-acylhydrolase (sscle_01g004820), and triacylglycerol lipase (sscle_01g008640) were significantly upregulated at the late stage of infection, specifically in the S line (Table S7). This suggests the induction of lipolytic enzymatic activity in *S. sclerotiorum* may depend on the immunity of the host. Lipases were also reported to act as virulence factors in the fungal phytopathogen *B. cinerea* [33], suggesting *S. sclerotiorum* lipases may play a role in virulence.

### Genes involved in the degradation of the host cell wall

As a necrotroph, degradation of the host cell wall is important during *S. sclerotiorum* infection to achieve the required plant cell death for growth and development [34]. A portion of the numerous cell wall degrading enzymes (CWDEs) identified in the *S. sclerotiorum* genome [15], including those involved in the degradation of lipids, cellulose, arabinogalactan, hemicellulose, mannan, pectin, starch and proteins, were upregulated during infection of chickpea (Table 2, Table S7). After breaching the cuticle, polygalacturonases (PGs) are often the first lytic enzymes produced by a pathogen [35, 36]. A putative exo-PG (sscle_05g046840, LogFC = 3.2–8.2) was the most upregulated relative to in vitro in the current study in both chickpea varieties relative to in vitro throughout the infection (Table S7). Four previously characterised PGs: endo-PGs *Sspg1* (sscle_16g108170) and *Sspg3* (sscle_09g070580), and exo-PGs *Ssxpg1* (sscle_02g018610) and *Ssxpg2* (sscle_04g035440) were also upregulated in the current study, relative to in vitro (Table S7). Infiltration of purified endo-PG into plant leaf tissues causes rapid loss of cell wall integrity followed by cell death, [37, 38] suggesting the importance of *Sspg1* and *Sspg3* in tissue maceration during *S. sclerotiorum* infection. Orthologs of *Ssxpg1* and *Ssxpg2* in *B. cinerea* (*BcPG1* and *BcPG2*) showed necrosis inducing activities, and disruption of either of the genes reduced virulence [28, 39], an indication of the significant role exo-PGs play in lesion development and host colonisation.

**Table 2:** The number of *in planta* upregulated *S. sclerotiorum* genes involved in the cell wall and cuticle degradation

Substrate	CWDE category	Number of upregulated genes in the category
Lipid/cutin	Cutin	14
Polysaccharides	Cellulose	19
	Arabinogalactan	6
	Hemicellulose	16
	Mannan	7
	Pectin	16
	Starch	3
Proteins/peptides	Protein	17

Proteases are hydrolytic enzymes that act as important virulence factors in many fungal plant pathogens by degrading host proteins that are involved in the immune response [40]. The *in planta* upregulation relative to in vitro of non-aspartyl acid protease (*acp1*; sscle_11g082980) was observed at all time points, peaking in expression at 24 hpi in both lines (LogFC = 7.2–7.9) (Table S7). Several factors control *acp1* induction, including glucose levels, nitrogen starvation and acidification [21]. Previous studies found upregulation of *acp1* at a later stage of *S. sclerotiorum* infection in *H. annuus* cotyledons [21], *G. max* petioles [23], and *B. napus* leaves [7], suggesting that *acp1* has a possible role in virulence on multiple plant species and that it responds to cues present at different infection stages in different hosts. Another gene encoding an aspartyl protease, sscle_07g058540, was upregulated at all stages of infection in the current study, with a peak expression relative to in vitro at 24 hpi (Table S7). The gene sscle_07g058540 is a homologue of several aspergillopepsin-like proteins (cd06097) in aspergillosis of humans, which act as a cofactor for the persistence of colonisation [41]. Putting this all together, sscle_07g058540 may be a catalyst that assists *S. sclerotiorum* growth and development during infection.

### *S. sclerotiorum* secondary metabolite synthesis and detoxification enzymes

Secondary metabolite (SM) polyketide synthases (PKSs) and non-ribosomal peptide synthases (NRPSs) were the major enzymes associated with SM synthesis in *S. sclerotiorum* and make up to 47.2% of the upregulated SM biosynthesis clusters in the current study (Table S8). The SM biosynthesis gene expressed at the highest level (LogFC = 7.6–9.2) was a gene encoding the PKS responsible for dihydroxy naphthalene (DHN) melanin biosynthesis (*PKS13*; sscle_03g031520) at 6–12 hpi as compared to the in vitro control, indicating a possible role in penetration during chickpea infection (Table S8). In a previous study, disruption of *S. sclerotiorum* genes involved in melanin biosynthesis showed no change in pathogenicity; however, slower development of mycelial and hyphal branching was observed [42]. The current results indicate the importance of melanin to aid appressoria mediated penetration of *S. sclerotiorum*.

Glutathione S-transferases (GSTs) play critical roles in the detoxification of xenobiotic chemicals in fungi by reducing them to glutathione [43]. The *S. sclerotiorum* GST most upregulated relative to in vitro in this study was a UDP-glucosyltransferase (*Ssbgt1*; sscle_01g003110, LogFC = 3.6–5.2) (Table 3). *Ssbgt1* plays a role in the degradation of the antimicrobial compound brassinin through glycosylation and is induced in response to the presence of a variety of plant phytoalexins [44].

**Table 3:** *Sclerotinia sclerotiorum* detoxification enzymes upregulated (LogFC) *in planta* relative to in vitro

		MR^a^ line hpi*					S^b^ line hpi*				
Gene ID	Description	6	12	24	48	72	6	12	24	48	72
sscle_01g003110	Glutathione S-transferase	3.6	3.5	4.9	3.6	4.1	–	2.5	5.2	3.1	3.8
sscle_01g005000	Glutathione S-transferase	–	–	3.7	3.1	2.9	–	–	3.2	–	–
sscle_08g067590	Glutathione S-transferase	–	–	–	2.8	2.9	–	–	–	–	–
sscle_02g021570	Laccase	4.3	–	–	–	–	4.6	4.5	–	–	–
sscle_01g005590	Cytochrome P450	–	–	–	3.3	2.9	–	–	–	2.9	3.1
sscle_04g033880	Cytochrome P450	4.1	4.2	5.6	4.9	5.9	–	2.2	2.3	2.6	5.7

^a^ moderately resistant line; ^b^ susceptible line; * hpi = hours post-inoculation

Other GSTs induced *in planta* in the current study were sscle_01g005000 (logFC = 2.9–3.7) in both lines and sscle_08g067590 (logFC = 2.8–2.9) in the MR line, at 24–72 hpi (Table 3). Disruption of GST genes *AbGSOT1* and *AbUre2pB1* in the host generalist brassica pathogen *Alternaria brassicicola* led to reduced virulence [45]. This indicates the importance of xenobiotic compound detoxification during fungal infection. The greatest upregulation of GSTs was observed in the MR line, possibly a reflection of host resistance exerted by the release of host defence-related antifungal compounds during infection.

Benzoic acid derivatives are aromatic compounds arising from the plant defence β- ketoadipate pathway [46]. The CYP enzyme, benzoate 4-hydroxylase, from *Aspergillus niger,* was reported to play a role in the hydroxylation of benzoic acid to 4-hydroxybenzoate [47]. An *S. sclerotiorum* CYP gene, sscle_01g005590, encoding benzoate 4-hydroxylase, was upregulated at 48–72 hpi in both chickpea lines (Table 3), which may suggest higher pressure from host defence toxins at the late stage during *S. sclerotiorum*-chickpea interaction.

### *Sclerotinia sclerotiorum* signalling pathways are vital during chickpea infection

A total of 24 *S. sclerotiorum* transcription factors were upregulated in the current study (Table S9). Two functionally characterised *S. sclerotiorum* TFs *Pac1* (sscle_06g049830) [48], and *Ssfkh1* (sscle_06g049780) [49] were upregulated *in planta*. *Pac1* was upregulated at 12 hpi in the S line only (LogFC = 2.8) during chickpea infection (Table S9). *Pac1* triggers oxalic acid (OA) biosynthesis in response to increased pH and reduces the ambient pH, which in turn causes an increase in *Sspg1* and *acp1* expression and promotes sclerotial development [48]. The upregulation of *Pac1* on the S line may suggest that S line tissues were more alkaline than those of the MR line.

Fungal histidine kinases play a vital role in controlling signalling pathways that regulate osmotic and oxidative stress responses, cell cycle control and virulence [15]. We found that the two-component sensor histidine kinase *Shk1* (sscle_16g107650) was upregulated at 12–72 hpi relative to inoculum in both lines (Table S9). A previous study showed disruption of *Shk1* led to reduced and altered hyphal growth and failed sclerotia formation [50]. Although *Shk1* mutants exhibited normal virulence, they showed sensitivity to osmotic stress and increased resistance to fungicides, which suggest that *Shk1* likely works upstream of the MAPK cascade to control these processes.

### A substantial portion of putative effectors are upregulated on both host varieties during infection

We compared the *S. sclerotiorum* expressed genes with the 523 secreted proteins identified in the *S. sclerotiorum* genome [13] to determine specific temporal changes in their regulation during chickpea infection. Of these, 173 were upregulated in both varieties, and 148 downregulated, with nine of the upregulated genes observed in the MR variety only and 27 in the S variety only (Table S10). Of the identified *S. sclerotiorum* secreted proteins, 78 were predicted to be candidate effectors by Guyon et al. [51] and 70 by Derbyshire et al. [13]. Of these candidate effectors, 32 were upregulated and 40 downregulated on both hosts during the current study (Table S11).

In addition to putative candidate effectors, we also considered the expression of experimentally characterised *S. sclerotiorum* effectors. Previous studies showed that *S. sclerotiorum* small cysteine-rich secreted protein *SsSSVP1* (sscle_01g003850) plays an essential role during infection by interfering with host respiration and inducing localised tissue necrosis [19]. In the current study, *SsSSVP1* was upregulated at 48 hpi in the MR line and 72 hpi in the S line (logFC = 5.1 and 5.7), respectively (Table S11). *S. sclerotiorum SsSSVP1* mutants showed reduced virulence in *B. napus* and *A. thaliana* [19]. Similarly, *SsSSVP1* upregulation was previously reported during the late stage of infection in *B. napus* [5, 17] and at all-time points in *G. max* [23]. The earlier induction *SsSSVP1* of in the MR line (48 hpi) compared to the S line (72 hpi) may suggest that temporal regulation of expression of *SsSSVP1* may depend on host susceptibility level.

Two *S. sclerotiorum* necrosis and ethylene-inducing (NEP) proteins (*SsNEP1* and *SsNEP2*) were characterised in a previous study on *Nicotiana benthamiana* and were reported to function as necrotrophic effectors [52]. The previous study showed upregulation of both genes at mid to later stages of infection with SsNEP2 expressed at a higher level than SsNEP1. In the current study, *SsNEP1* (sscle_04g039420) was not differentially expressed, and *SsNEP2* (sscle_12g090490) was upregulated at the later stages of infection (48 hpi in MR and at 48–72 hpi In S lines) relative to in vitro (Table S11). Orthologs of these two genes in *B. cinerea* (*BcNEP1* and *BcNEP2*) are both proteins capable of inducing necrosis in dicotyledonous but not monocotyledons host [53].

### Expression of genes related to oxalic acid production and reactive oxygen species regulation

Oxalic acid (OA) has roles in weakening the host cell wall, activating hydrolytic enzymes, suppressing the oxidative burst and intensifying programmed cell death (PCD), leading to full colonisation [54]. A gene encoding oxalate decarboxylase (*Ss-odc2*: sscle_09g069850) was highly upregulated at the very early stage (6–24 hpi) of infection (LogFC = 6.5–8.4) and showed lower expression at the later stage (LogFC = 3.8–4.2) of infection relative to in vitro, in both chickpea lines (Table 4). *Ss-odc2* protects the pathogen cells by preventing excess accumulation of OA [55]. A previous study suggested that an alternative route of OA biosynthesis may be utilised during *S. sclerotiorum* early stages of infection [56]. Expression of *Ss-odc2* without the induction of *Ssoah1* in the current study may support previous findings that OA is not the only source of acidification or determinant of *S. sclerotiorum* virulence expression [57], or alternatively, the host tissue was already acidic enough for growth.

**Table 4:** *Sclerotinia sclerotiorum* reactive oxygen scavenging (ROS) enzymes upregulated (LogFC) during chickpea infection relative to in vitro

		MR^a^ line hpi*					S^b^ line hpi*				
Gene ID	Description	6	12	24	48	72	6	12	24	48	72
sscle_15g104430	Catalase	–	–	–	5.1	5.7	–	–	–	–	4.5
sscle_04g037170	Catalase	–	–	–	5.6	5.8	–	–	–	–	4.7
sscle_03g026200	Catalase	–	–	–	–	–	–	–	–	–	2.9
sscle_09g069850	Oxalate decarboxylate	8.4	6.5	–	4.1	4.2	8.2	8.1	–	–	3.8
sscle_04G035020	Peroxidase	–	3.5	3.5	4.3	5.1	–	–	5.3	5.4	4.7
sscle_15g102360	Peroxidase	3.6	–	–	–	–	4.1	3.8	–	–	–
sscle_09g070530	Peroxidase	4.1	–	–	–	–	–	3.6	–	–	3.6
sscle_08g065740	Peroxidase heme-thiolate	4.5	–	–	–	–	4.6	–	–	–	–

^a^ moderately resistant line; ^b^ susceptible line; * hpi = hours post-inoculation

An *S. sclerotiorum* gene, sscle_09g069850, with a bicupin domain, was previously reported to be a possible oxalate oxidase enzyme [58]. This gene was highly upregulated at 6–12 hpi (logFC = 7.6–8.7) and expression decreased at 48–72 hpi (logFC = 3.8–4.2) with no expression at 24 hpi, in both chickpea lines, relative to in vitro (Table 4). A previous study suggested that oxalate oxidases play a role in inducing programmed cell death [54]. The pattern of sscle_09g069850 expression in the current study may suggest involvement in both the biotrophic and necrotic stages during chickpea interaction.

Catalases and peroxidases are also important *S. sclerotiorum* ROS scavengers [59]. Three catalases, sscle_03g026200 (*Sscat1*), sscle_04g037170, and sscle_15g104430, were upregulated during the late stage of infection (48–72 hpi) in the MR and 72 hpi in the S line (Table 4). The most upregulated catalase during the current study was the previously characterised *Scat1* (sscle_04g037170). *Scat1* mutants show slower radial growth, a higher number of small sclerotia and hypovirulence [60]. The upregulation of catalases and peroxidases was observed at an early stage in the MR line and later stage in the S line during infection, suggesting *S. sclerotiorum* induces ROS scavengers depending on the host speed of employing defence responses.

## Conclusion

Our study demonstrates a continuum of activities that occurs during infection and colonisation of *C. arietinum* by *S. sclerotiorum*. In support of our study hypothesis, we observed significant upregulation of *S. sclerotiorum* genes *in planta* irrespective of the host’s susceptibility level. To our knowledge, this is the first RNA-seq study to investigate *in planta* gene expression in *S. sclerotiorum* during *C. arietinum* infection. The current findings showed that *S. sclerotiorum* induced numerous virulence factors, including CAZymes, transportation enzymes, detoxification enzymes, metabolites and putative secreted effector proteins during penetration and subsequent proliferation through the host.

In conclusion, the present study provides an insight into global transcriptional changes in *S. sclerotiorum* during infection of chickpea varieties differing in their susceptibility to the pathogen. Our findings further emphasise the role of CAZymes and proteases, in addition to secreted effectors, transporters and detoxifying enzymes during the growth and development of *S. sclerotiorum* within chickpea plants. Temporal changes in expression have demonstrated that *S. sclerotiorum* specific gene expression may depend on host susceptibility level. Detailed investigation of the identified genes could elucidate their precise roles and to determine if they represent viable targets for disease management.

## Material and methods

### Plant material

Two chickpea desi varieties with different levels of susceptibility to *S. sclerotiorum* were used as hosts in this study [61]. Seeds of moderately resistant (PBA HatTrick) and highly susceptible (Kyabra) chickpea varieties were planted in 5 cm pots with an all-purpose potting mix (UWA mix, Richgro, Perth, Australia) and grown for 8 weeks in a plant growth chamber with a 16-h photoperiod, a 22/16 °C day/night temperature, and 60% relative humidity. Once germinated, seedlings were watered as necessary and fertilised at 4 weeks with Nitrophoska perfekt™ fertiliser (Incitec Pivot fertilisers, Victoria, Australia). These two varieties are herein referred to as the MR line (moderately resistant PBA HatTrick) and S line (susceptible Kyabra).

### Fungal material preparation and inoculation

An aggressive *S. sclerotiorum* isolate CU8.20 was previously found to be aggressive in *B. napus* [62] and chickpea [61]. The isolate CU8.20 culture was prepared from dry sclerotia, which were cut in half and placed mycelium-side down on potato dextrose agar (PDA) (Becton Dickinson, USA) and incubated at 20 °C for 5–7 days in the dark plates. Subsequently, a mycelial plug was cut from actively growing edges of the PDA culture and sub-cultured in a fresh PDA plate at 20 °C for two days. Only plates with consistent mycelial growth were used for inoculation. Eight-week-old plants were infected following a stem inoculation assay that involved cutting a 5 mm plug from the actively growing mycelium and placing it in the middle of the plant stem. The plug was secured using Parafilm® to maintain moisture. The in vitro control samples were generated by inoculating potato dextrose broth (PDB) with 5 mm minimal media agar plugs and incubated at 26 °C with shaking at 160 rpm for 96 h.

### Sample collection

*S. sclerotiorum* mycelium (in vitro samples) was collected from PDB and flash-frozen in liquid nitrogen. Inoculated plant stem sections were collected by cutting the stem 1 cm above and below the point of inoculation or the lesion at six, 12, 24, 48, and 72 h post-inoculation (hpi), immediately put in an Eppendorf tube and flash-frozen in liquid nitrogen within 10 s of collection and stored at − 80 °C until RNA extraction. Each treatment (time point /variety) consisted of three biological replicates. Six stem sections were collected from four individual plants and pooled for one biological replicate.

### RNA extraction and sequencing

The fungal mats and the infected stem samples were ground into a fine powder with liquid nitrogen pre-cooled in an RNAse-free mortar and pestle. Total RNA was extracted from inoculated chickpea stem tissues and *S. sclerotiorum* mycelium following the Trizol™ Reagent protocol (Invitrogen Corp., Carlsbad, CA, USA). RNA quantity and quality were assessed using the Qubit fluorometry assay (Invitrogen Corp., Carlsbad, CA, USA). Novogene performed library preparation (150 bp paired-end) and sequencing on an Illumina HISEq 2500 platform.

### RNA sequencing data quality control

Quality assessment on raw fastq reads and cleaned reads was conducted using the FastQC tool (V. 0.11.8) (www.bioinformatics.babraham.ac.uk/projects/fastqc). Trimmomatic v.0.38 was used to trim low-quality base calls, and filter adapters and low quality reads [63]. The following trimmomatic parameters were used, ILLUMINACLIP: TruSeq3-SE: 2:30:10 MINLEN: 36 LEADING: 3 TRAILING: 3 SLIDINGWINDOW: 4:15.

### Read alignment

The trimmed reads were split between the pathogen (*S. sclerotiorum* strain 1980 genome, Bioproject PRJNA348385, assembly ASM185786v1) [13] and the host (*Cicer arietinum,* Bioproject PRJNA190909, assembly ASM33114v1) [64] using BBSplit tool v.38.12 (https://sourceforge.net/projects/bbmap/). Quality filtered *S. sclerotiorum in planta* and in vitro reads were aligned to the reference genomes using HISat2 v2.1.0 [65]. The resulting alignments in SAM format were then converted to BAM format, sorted and indexed using SAMtools v.0.1.19 [66]. The number of reads that mapped to each gene in the reference (read counts/gene counts) were generated using HTseq package v 0.12.4 [67].

### Differential gene expression analysis

The *S. sclerotiorum in planta* differential gene expression analysis was conducted using edgeR and limma Bioconductor packages in R v4.0.2 [68, 69]. The raw count data were normalised using the Trimmed Mean of M-values (TMM) method. Principle coordinate analysis (PCoA) plots were generated using the *plot-MDS ()* function from limma and the *heatmap.2 ()* function from gplots to determine the relatedness of the biological replicates. Pairwise contrasts were performed using quasi-likelihood F tests [70]. A false discovery rate (FDR) cut-off of 0.05 was applied, and a log_2_ fold change cut-off of ≥2 to indicate upregulation and ≤ − 2 to indicate down-regulation. Differentially expressed genes (DEGs) were considered at each time point for each host in relation to the in vitro/vegetative growth culture (hypothetical time-point 0).

### Gene ontology enrichment analysis of DEGs

*S. sclerotiorum* Gene ontology (GO) terms were derived from a previous InterPro annotation [13]. To test for significantly enriched gene ontology (GO) categories, we used the R Bioconductor package TopGO (2007) to implement the classical method and Fisher’s exact test with a *P*-value threshold of ≤0.05 [71]. GO terms for the full *S. sclerotiorum* total gene list were used as the background list for enrichment analysis.

### CAZymes and secreted protein effector analysis

CAZymes were predicted using DBCan2 web server v8.0 [72]. The *S. sclerotiorum* strain 1980 protein sequences were used as the input for CAZymes prediction (GCA_001857865) [13]. The CAZymes prediction was made using three databases, HMMER, DIAMOND and Hotpep [72]. Only those CAzymes identified by at least two databases and with positive SignalP scores were considered for analysis. This study considered previously predicted effectors by Derbyshire et al. [13] and Guyon et al. [51].

### Validation of RNA-seq data using reverse transcription-quantitative PCR

RNA-Seq data was validated by performing reverse transcription quantitative polymerase chain reactions (RT-qPCRs) on five upregulated genes and one downregulated gene. Two-time points were chosen to represent the early stage (12 hpi) and late stage (48 hpi) of MR and S line infection. RNA samples used for qPCR validation were the same samples used for Illumina sequencing. The RNA samples were reverse transcribed using the First-Strand cDNA Synthesis Kit for RT-PCR (AMV) (NEB Inc. Ipswitch, MA) according to the manufacturer’s instruction. The cDNA samples were then diluted 1:20 before qPCR. Real-time quantification for MR line was performed with iTaq Universal SYBR Green Supermix and S line with PowerUp SYBR Green master mix with the following cycling conditions: 95 °C for 2 min, then 95 °C for 15 s, 60 °C for 30 s and 72 °C for 15 s, repeated 40 cycles, followed by 72 °C for 2 min. The relative expression of genes was calculated using the 2^- ∆∆Ct^ method [73] with the fungal β-tubulin gene (sscle_02g015170) used as an endogenous control. Three biological replicates were used, and three technical replicates per biological replicate to determine the expression levels for the six genes relative to fungal β-tubulin. The qPCR experiment consisted of three biological replicates per sample and three technical replicates per biological replicate.

## Supplementary Information

**Additional file 1: Table S1.** Primers used in the validation of RNA sequencing data.

**Additional file 2: Table S2**. List of differentially expressed *S. sclerotiorum* genes during the interaction with moderately resistant (MR) and susceptible (S) chickpea lines at 6, 12, 24, 48 and 72 hours post inoculation ( P. Adj. <0.05; LogFC ≥2 indicated upregulated or ≥2 indicate downregulated.

**Additional file 3: Table S3**. List of common *S. sclerotiorum* genes differentially expressed during the interaction with moderately resistant (MR) and susceptible (S) at 6, 12, 24,48 and 72 post inoculation relative to *in vitro*.

**Additional file 4: Table S4**. List *S. sclerotiorum* genes differentially expressed exclusively in moderately resistant (MR) only and susceptible (S) only at 6, 12, 24,48 and 72 post inoculation relative to *in vitro* during chickpea infection

**Additional file 5: Table S5**. Enrichment analysis of *S. sclerotiorum* upregulated genes during interaction with moderately resistant (MR) and susceptible (S) chickpea lines at 6, 12, 24, and 72 hours post inoculation relative to *in vitro* control.

**Additional file 6: Table S6**. Enrichment analysis of *S. sclerotiorum* downregulated genes during interaction with moderately resistant (MR) and susceptible (S) chickpea lines at 6, 12, 24, and 72 hours post inoculation relative to *in vitro* control.

**Additional file 7: Table S7**. Description of temporal *S. sclerotiorum in planta* upregulated genes involved in cell wall degradation during chickpea infection relative to *in vitro* control (P. Adj. <0.05; LogFC ≥ 2).

**Additional file 8: Table S8**. Secondary metabolite synthesis, cytochrome P450 and transporter genes upregulated at some timepoint of the MR and S lines infection in comparison to *in vitro* control ( P. Adj. <0.05; LogFC ≥ 2).

**Additional file 9: Table S9.** *S. sclerotiorum* transcriptional factors which were upregulated at some timepoint of the MR and S lines infection in comparison to *in vitro* control ( P. Adj. <0.05; LogFC ≥ 2).

**Additional file 10: Table S10.** Secreted proteins upregulated and downregulated during the course of MR and S lines infection.

**Additional file 11: Table S11.** Predicted *S. sclerotiorum* putative effector candidate’s upregulated at some timepoint MR and S lines infection relative to *in vitro* control (P. Adj. <0.05; LogFC ≥ 2).

**Additional file 12: Figure S1:** Differentially expressed genes in MR and S line at 6, 12, 24, 48 and 72 hpi based on expression pattern relative to *in vitro* (P. Adj. < 0.05; LogFC upregulated ≥ 2 and downregulated ≤ 2).The colours indicate the fold change with red = upregulated, black = regulated and green = downregulated genes.

**Additional file 13: Figure S2:** Differentially expressed genes exclusively in MR only and S line only at 6, 12, 24, 48 and 72 hpi based on expression pattern relative to *in vitro* (P. Adj. < 0.05; LogFC upregulated ≥ 2 and downregulated ≤ 2).The colours indicate the fold change with red = upregulated, black = regulated and green = downregulated genes.

**Additional file 14: Figure S3:** Heatmap showing the top 20 GO categories of upregulated genes at 6, 12, 24, 48 and 72 hpi in a moderately resistant (MR) and susceptible (S) chickpea lines based on –log(10)fold change (enrichment). The colours indicate the enrichment with green = high enrichment, and red = low enrichment.

**Additional file 15: Figure S4:** Heatmap showing the top 20 GO categories of downregulated genes at 6, 12, 24, 48 and 72 hpi in a moderately resistant (MR) and susceptible (S) chickpea lines based on –log(10)fold change (enrichment). The colours indicate the enrichment with green = high enrichment, and red = low enrichment.

## Data Availability

The raw data used in this study has been deposited to the sequence read archive under BioProject ID: PRJNA687280. The gene identifiers prefixed with ‘sscle_’ used throughout this manuscript are locus tags of *S. sclerotiorum* proteins that are available in the NCBI protein database.

## Abbreviations

CAZymes: Carbohydrate-active enzymes
CWDE: Cell wall-degrading enzymes
CYP450: Cytochrome 450
DEG: Differentially expressed genes
FDR: False discovery rate
GO: Gene ontology
GST: Glutathione S – transferase
LogFC: Log_2_ fold change
MR: Moderately resistant
OA: Oxalic acid
PCD: Programmed cell death
PDA: Potato dextrose agar
PG: Polygalacturonase
PKS: Polyketide synthase
ROS: reactive oxygen species
S: Susceptible
SM: Secondary metabolites
SSR: Sclerotinia stem rot
TF: Transcription factor
