# Supplementary figures and images for: Analysis of differentially expressed Sclerotinia sclerotiorum genes during the interaction with moderately resistant and highly susceptible chickpea lines
Source: BMC Genomics. 2021 May 8;22:333. doi: 10.1186/s12864-021-07655-6 (PMC8106195; doi:10.1186/s12864-021-07655-6)

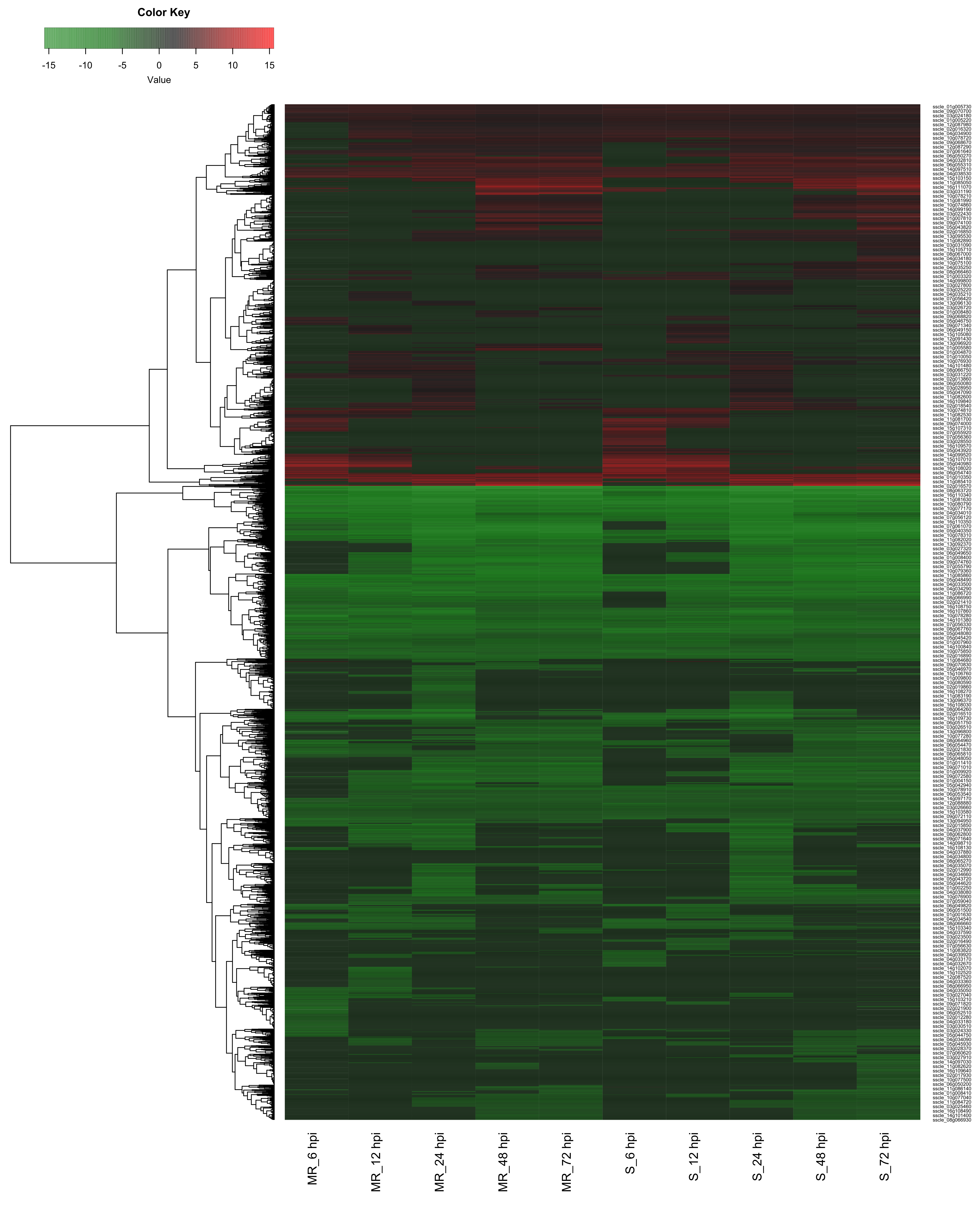

Supplement: Supplementary file 12 — Additional file 12: Figure S1: Differentially expressed genes in MR and S line at 6, 12, 24, 48 and 72 hpi based on expression pattern relative to in vitro (P. Adj. < 0.05; LogFC upregulated ≥ 2 and downregulated ≤ 2).The colours indicate the fold change with red = upregulated, black = regulated and green = downregulated genes. [file 12864_2021_7655_MOESM12_ESM.png]

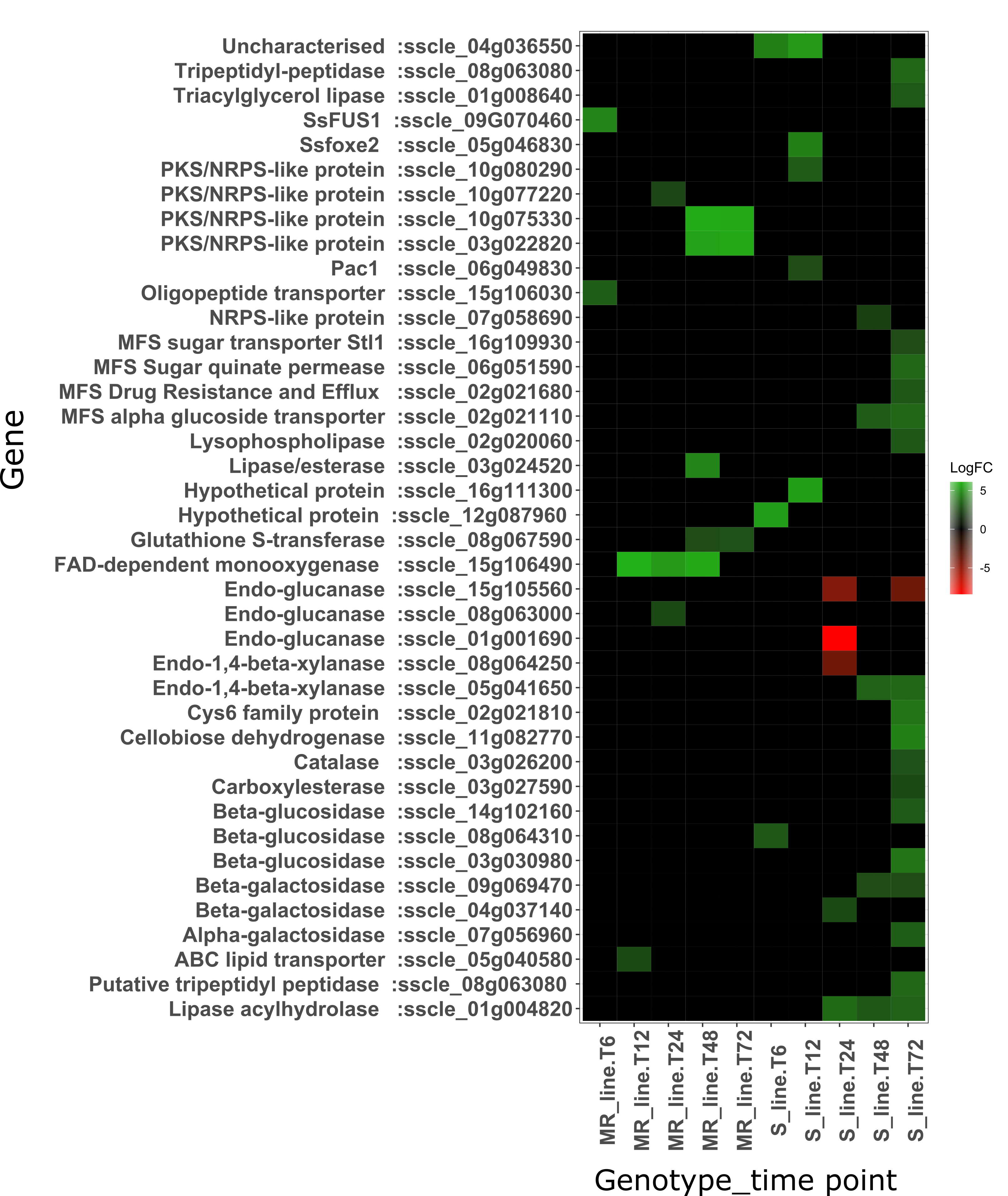

Supplement: Supplementary file 13 — Additional file 13: Figure S2: Differentially expressed genes exclusively in MR only and S line only at 6, 12, 24, 48 and 72 hpi based on expression pattern relative to in vitro (P. Adj. < 0.05; LogFC upregulated ≥ 2 and downregulated ≤ 2).The colours indicate the fold change with red = upregulated, black = regulated and green = downregulated genes. [file 12864_2021_7655_MOESM13_ESM.png]

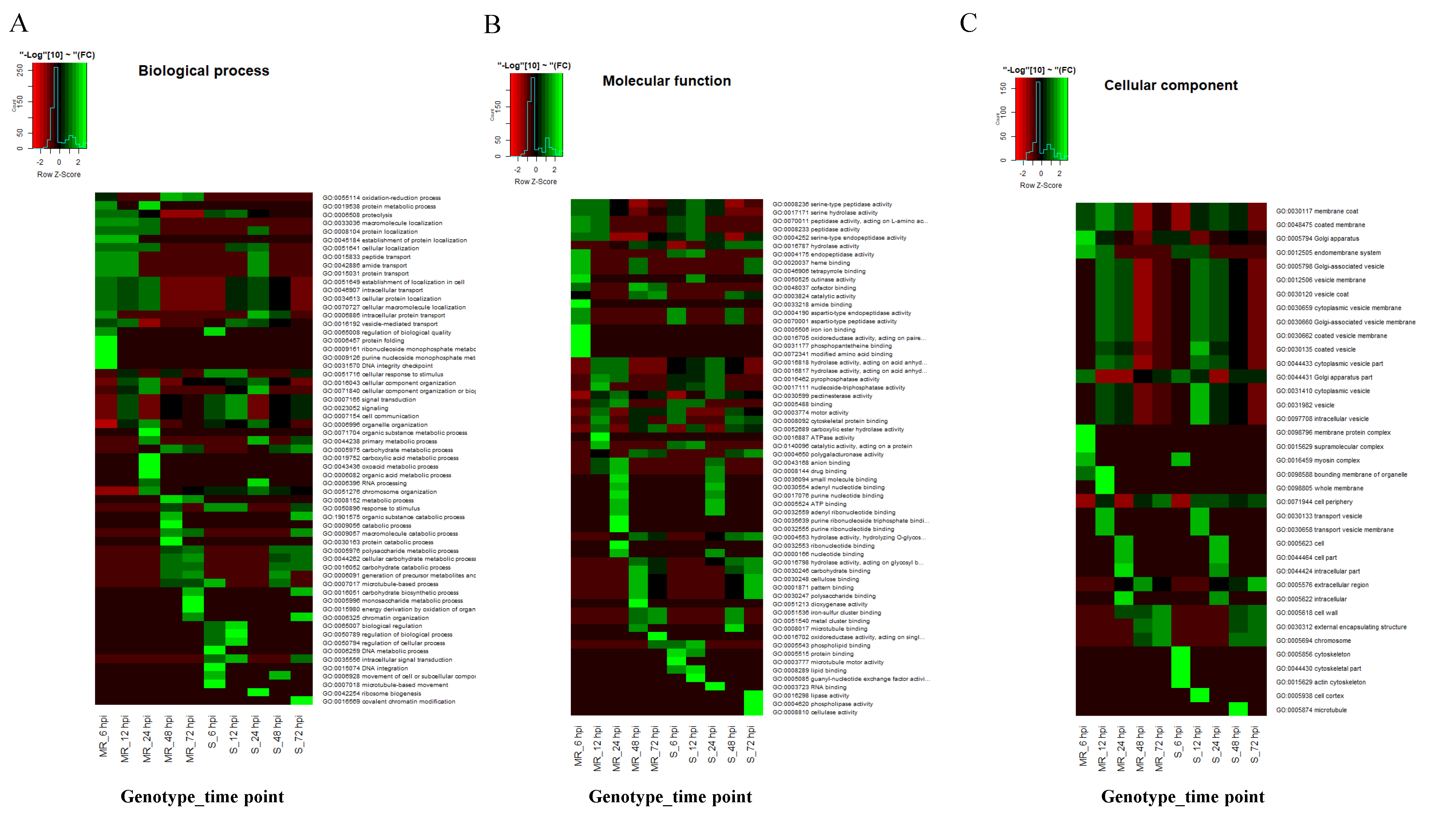

Supplement: Supplementary file 14 — Additional file 14: Figure S3: Heatmap showing the top 20 GO categories of upregulated genes at 6, 12, 24, 48 and 72 hpi in a moderately resistant (MR) and susceptible (S) chickpea lines based on –log(10)fold change (enrichment). The colours indicate the enrichment with green = high enrichment, and red = low enrichment. [file 12864_2021_7655_MOESM14_ESM.png]

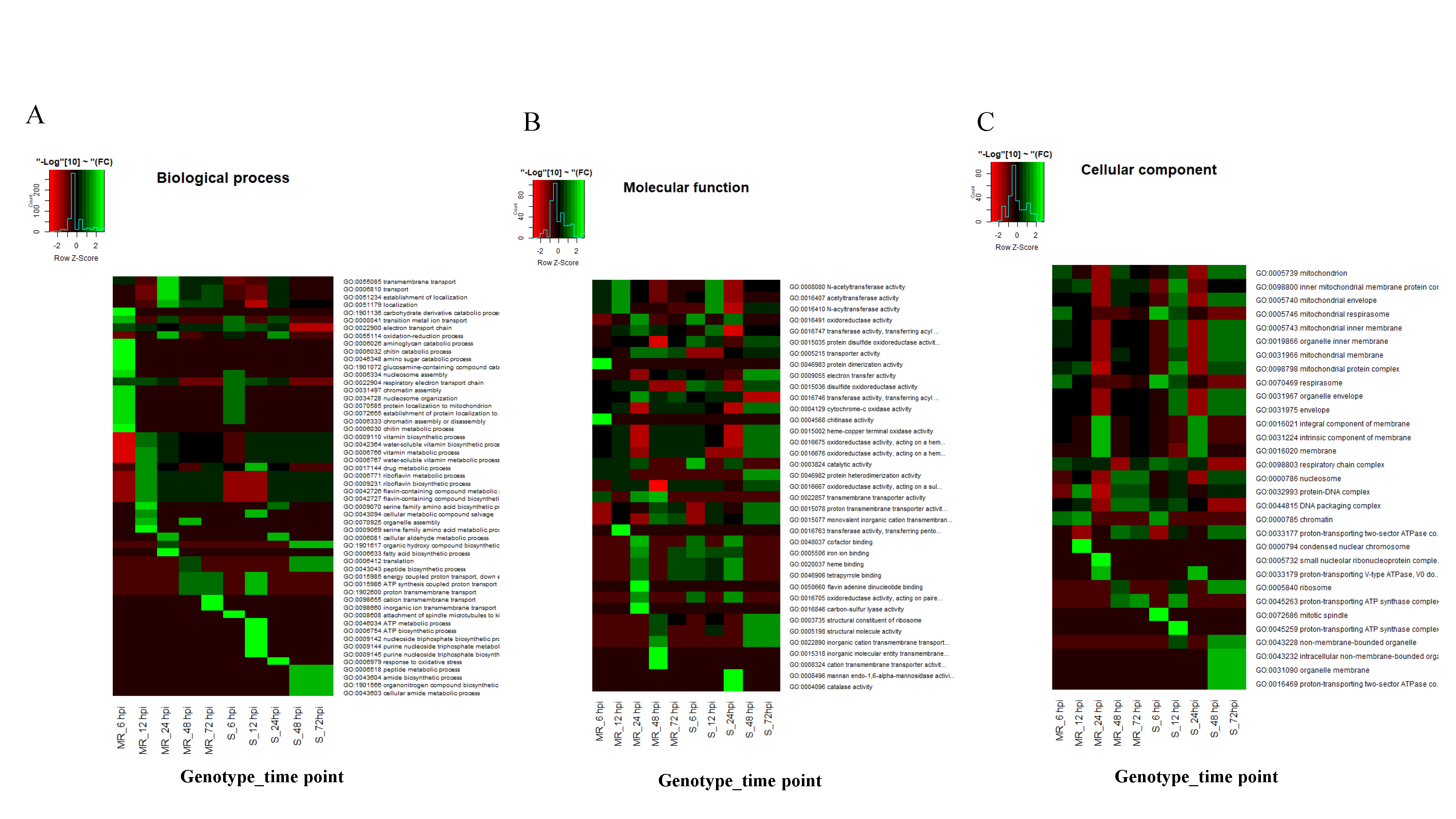

Supplement: Supplementary file 15 — Additional file 15: Figure S4: Heatmap showing the top 20 GO categories of downregulated genes at 6, 12, 24, 48 and 72 hpi in a moderately resistant (MR) and susceptible (S) chickpea lines based on –log(10)fold change (enrichment). The colours indicate the enrichment with green = high enrichment, and red = low enrichment. [file 12864_2021_7655_MOESM15_ESM.png]
